# Supplementary material for: Multi-disciplinary Evaluation of Sexual Assault Referral Centres (SARCs) for better Health (MESARCH): protocol for a 1-year cohort study examining health, well-being and cost outcomes in adult survivors of sexual assault attending SARCs in England
Source: BMJ Open. 2022 May 23;12(5):e057449. doi: 10.1136/bmjopen-2021-057449 (PMC9131084; doi:10.1136/bmjopen-2021-057449)

BMJ Open

BMJ Open is committed to open peer review. As part of this commitment we make the peer review history of every article we publish publicly available.

When an article is published we post the peer reviewers' comments and the authors' responses online. We also post the versions of the paper that were used during peer review. These are the versions that the peer review comments apply to.

The versions of the paper that follow are the versions that were submitted during the peer review process. They are not the versions of record or the final published versions. They should not be cited or distributed as the published version of this manuscript.

BMJ Open is an open access journal and the full, final, typeset and author-corrected version of record of the manuscript is available on our site with no access controls, subscription charges or pay-per-view fees (http://bmjopen.bmj.com).

If you have any questions on BMJ Open's open peer review process please email info.bmjopen@bmj.com

BMJ Open

Multi-disciplinary Evaluation of Sexual Assault Referral Centres (SARCs) for better Health (MESARCH): Protocol for a one-year cohort study examining health, wellbeing and cost outcomes in adult survivors of sexual assault attending SARCs in England

Journal:	*BMJ Open*
Manuscript ID	bmjopen-2021-057449
Article Type:	Protocol
Date Submitted by the Author:	15-Sep-2021

Complete List of Authors:	O'Doherty, Lorna; Coventry University, Faculty of Health and Life Sciences<br>Carter, Grace; Coventry University<br>Musto, Alex; Coventry University<br>Caswell, Rachel; University Hospitals Birmingham NHS Foundation Trust, Rachel Caswell<br>Jackson, Louise; University of Birmingham, Health Economics Unit<br>Feder, Gene; University of Bristol, Community based medicine<br>Morris, Richard; University of Bristol, School of Social & Community Medicine<br>Brown, Katherine; University of Hertfordshire, Department of Psychology, Sports Science and Geography

Keywords:	SEXUAL MEDICINE, PUBLIC HEALTH, MENTAL HEALTH, HEALTH SERVICES ADMINISTRATION & MANAGEMENT

SCHOLARONE™
Manuscripts

Multi-disciplinary Evaluation of Sexual Assault Referral Centres (SARCs) for better Health (MESARCH): Protocol for a one-year cohort study examining health, wellbeing and cost outcomes in adult survivors of sexual assault attending SARCs in England

Lorna O'Doherty[1], Grace Carter[1], Alex Musto[1] Rachel Caswell[2], Louise Jackson[3], Gene Feder[4], Richard Morris[4], & Katherine E Brown[5]

1. Centre for Intelligent Healthcare, Faculty of Health & Life Sciences, Coventry University, Priory St, Coventry CV1 5FB.
2. Department of Sexual Health and HIV Medicine, University Hospitals Birmingham, Birmingham, B15 2TH
3. Health Economics Unit, Institute of Applied Health Research, University of Birmingham, Birmingham, B15 2TT
4. Centre for Academic Primary Care, Population Health Sciences, Bristol Medical School, University of Bristol, Bristol, UK
5. Department of Psychology, Sports Science and Geography, School of Life and Medical Sciences, University of Hertfordshire, Hatfield, AL10 9AB

Corresponding author: Professor Katherine Brown

Department of Psychology, Sports Science and Geography, School of Life and Medical Sciences, University of Hertfordshire, Hatfield, AL10 9AB

Email: k.brown25@herts.ac.uk

Word count: 5032

For peer review only

For peer review only - http://bmjopen.bmj.com/site/about/guidelines.xhtml

ABSTRACT

Introduction.

Sexual violence is commonplace and has serious adverse consequences for physical and mental health. Sexual Assault Referral Centres (SARCs) are viewed as a best practice response. Little is known about their effectiveness and cost-effectiveness. Long-term data on the health and wellbeing of those who have experienced rape and sexual assault are also lacking.

Methods and analysis.

This is a protocol for a one-year cohort study examining health and wellbeing in survivors of sexual violence recruited via SARCs in England. Measures are being taken at baseline, 6, and 12 months. Post-traumatic stress (PTS) is the primary outcome (target N=270 at 12-month follow-up). Secondary measures include anxiety, depression, substance use and sexual health and wellbeing. Our analysis will examine whether variation in SARC service delivery is associated with different trauma symptoms at 12 months. Where sub-groups are large enough, we will explore differences in outcome by characteristics such as gender and ethnicity. An economic analysis will compare costs and outcomes associated with different organisational aspects of SARC service delivery and levels of satisfaction with care. A nested qualitative study will employ Narrative Analysis of transcribed interviews with 30 cohort participants and 20 survivors who have not experienced SARC services.

Ethics and dissemination.

The research is supported by an independent study steering committee (SSC), data monitoring and ethics committee (DMEC) and Patient and Public Involvement (PPI) group. A central guiding principle of the research is that being involved should feel like the opposite of being a victim of sexual violence; empowering and supportive. Our PPI representatives are instrumental in this, and our wider stakeholders are helping us consider the health and wellbeing of all involved. We will disseminate widely through peer-reviewed articles and non-academic channels to maximise the impact of findings on commissioning of services and support for survivors.

ISRCTN registry: https://doi.org/10.1186/ISRCTN30846825

ARTICLE SUMMARY

Strengths and limitations of this study

For peer review only

For peer review only - http://bmjopen.bmj.com/site/about/guidelines.xhtml

- The study proposes the most substantive investigation to date in the UK of health, wellbeing and service utilisation in survivors of sexual assault and rape
- The study applies a longitudinal design and nationally representative sample of adults taking up SARC services following experience of sexual assault or rape, including all genders to look at outcomes over one year
- The study considers a wider definition of sexual health than just STI acquisition
- The study uses innovative approaches to recruitment that acknowledge barriers to research participation affecting the target population and is supported by a wide range of stakeholders nationally including a group of people with lived experience of sexual abuse
- Relatively few survivors of sexual assault and rape have used SARCs, so generalisability to all people who have experienced sexual violence and abuse may be limited

For peer review only

For peer review only - http://bmjopen.bmj.com/site/about/guidelines.xhtml

INTRODUCTION

Sexual violence is commonplace, with evidence suggesting that one in five women and one in twenty-five men in England and Wales have experienced sexual assault since the age of 16 [1]. Sexual violence refers to any sexual act, or attempt at a sexual act, or an act directed at a person's sexuality, involving coercion [2]. Sexual violence includes but is not restricted to: rape, sexual assault, child sexual abuse, sexual harassment, rape within marriage and relationships, forced marriage, so-called honour-based violence, female genital mutilation, trafficking, sexual exploitation, and ritual abuse [2]. Sexual violence often goes unreported to the police, but arguably, recent shifts in the public understanding of sexual violence and developments that include the *#metoo* movement on social media have likely been contributing to year-on-year increased rates of reporting [3]. In the year to end of March 2017, one in six people reported their experience to the police - 121,187 incidents of sexual assault in total, representing a 14% increase on the previous year [3].

The serious and devastating effects of sexual violence are a well-documented public health burden [4]. They include a range of immediate and long-term physical and mental health consequences. For women who have experienced sexual violence, physical health consequences include unwanted pregnancy, sexually transmitted infections (STIs) [4], urinary tract infections, painful sex, chronic pelvic pain and vaginal bleeding [5]. For male victims, physical health consequences include genital and rectal injuries and erectile dysfunction [6]. The mental health sequelae of sexual violence has been found to be equally substantial across different population groups [4 6-8]. Post-traumatic stress (PTS) is common amongst those who have experienced sexual violence [9 10] and its incidence and severity are similar in both men and women [11]. Other mental health consequences include alcohol use disorders, eating disorders, anxiety, depression, self-harm and suicidality [4]. In addition, experiencing mental health problems such as lifetime PTS is associated with increased risk of other long-term health conditions including hypertension, cardiovascular disease and gastrointestinal problems [9]. Thus, sexual violence produces significant health burden, with each experience of rape in adults estimated to cost over £73,000 in psychological and physical impacts of injuries/illnesses and resultant health service use alone [12]. The wider economic costs to society reach over £8billon per year [12].

Given the substantial personal, societal and economic costs of sexual violence, an effective and consistent response for survivors is imperative. In the 2003 guidelines on the provision of care for victims of sexual violence, the World Health Organization recommended that an initial response to survivors should include medico-legal and health services provided at the same time, in the same location and preferably by the same health practitioner. Policy-

For peer review only - http://bmjopen.bmj.com/site/about/guidelines.xhtml

makers, commissioners and health workers have been encouraged to develop this model of service provision [13]. In the USA for example, many States offer Sexual Assault Nurse Examiner (SANE) programs or Sexual Assault Response Teams (SARTs) to provide these recommended services. In England and other parts of the United Kingdom, there has been a steady growth in Sexual Assault Referral Centres (SARCs) as a best practice response for survivors after incidents of sexual assault or rape [14]. SARCs, as a form of medico-legal and health service, are intended to co-ordinate all of the care and support needs for survivors. This may include immediate post-incident crisis support, forensic medical examination (FME) for the purposes of collecting evidence needed to prosecute perpetrators, referral to sexual health and other healthcare services, referral for counselling and the provision of an independent sexual violence advisor (ISVA) for ongoing support, particularly if that includes dealing with the criminal justice system. At the time of writing, there are reportedly 47 SARCs in England including a number of specialist paediatric sites [15], operating with considerable variation in their service models [16]. Currently, little is known about the effectiveness and cost-effectiveness that the different service models may represent in addressing the physical and mental health outcomes for survivors of sexual violence outlined above. In addition, although the substantial negative impacts of sexual violence are well-documented [4] much less is known about the longer-term health and wellbeing outcomes for survivors, particularly in relation to the model of care they may have received. Data on different population sub-groups whose risk is increased due to social inequities and marginalisation (e.g. from members of migrant, ethnic minority, and sexual and gender minority communities) has also been identified as limited. Furthermore, there is a lack of data on the experiences of male survivors [17]; those with a disability even though they are at greater risk of sexual violence [18]; interactions between sexual violence exposure and chronic mental health problems, and co-morbidity of health outcomes, non-partner sexual violence [19] and sexual health outcomes other than the acquisition of STIs [20].

This mixed-methods cohort study is the first to consider the health outcomes and cost trajectories of those who attend SARCs in England. It aims to evaluate: outcomes for different interventions and SARC models; outcomes for different subgroups of survivors attending SARCs; and outcomes from different post-crisis mental health care. It represents the first such study to consider the broader sexual health and wellbeing of survivors attending SARCs alongside other assessments of their health and mental wellbeing over time. We also report here how findings from our pilot study at one English SARC and our early data collection experiences have influenced a revised research protocol.

For peer review only - http://bmjopen.bmj.com/site/about/guidelines.xhtml

METHODS AND ANALYSIS

Design and setting

This is a cohort study of mental, physical, sexual health and cost outcomes over one year in adult survivors of sexual assault and rape who have received care through SARCs in England. Measures are taken at baseline (shortly after an index SARC visit) and at 6 and 12-months post-baseline. The study is part of the MESARCH programme currently funded from September 1, 2018 to May 31, 2022. A nine-month no cost extension is currently being considered by the funder.

Sampling, recruitment and procedures

We have recruited a stratified random sample of 15 of all the current practicing adult SARCs in England (47 identified at time of writing) with strata defined according to service delivery model, size and level of integration of services. To do this, we have drawn on national indicators of performance data collected by NHS England [14], the commissioning body for SARCs, and data collected from SARCs as part of the wider MESARCH study.

Recruitment is taking place in three phases. Firstly, over 25 months (August 2019-September 2021), service users aged 18 years and above presenting in person at any of the 15 participating SARCs have been screened. Due to changes required to services because of the Covid-19 pandemic, we are also including those whose only contact with a participating SARC is remote but who would have normally been seen face-to-face pre-Covid. People are excluded if in exercising judgement, the responsible member of SARC staff conducting screening, anticipates they may encounter difficulties in providing informed consent due to severe mental health issues, cognitive impairment or intellectual disability. We also exclude those visiting from prison settings, as conducting follow up assessments in prisons is outside the scope of the project. SARC staff complete a screen form for all those over 18 presenting to the service, recording basic demographic data, and offence and referral characteristics and indicating whether the person is eligible or not, with any reasons for exclusion noted. Screen forms, which at this stage contain pseudonymised/de-identified information about eligible and ineligible service users, are then passed using a secure web-based platform hosted by the University to the research team, providing comprehensive information about the pool of service users from which participants will be drawn.

The second phase of recruitment involves onward referral settings. Because many SARCs refer on quickly to other voluntary sector agencies, for each participating SARC, we have identified all linked support agencies and have worked to engage them. In situations where eligibility assessment has been completed by the SARC and the person has been referred

For peer review only - http://bmjopen.bmj.com/site/about/guidelines.xhtml

on to support in the voluntary sector, the voluntary agency take responsibility for the next stage of the recruitment process - seeking consent to pass service user contact details to the research team. Either SARC staff or voluntary agency staff then complete a consent to be contacted form for the individual service user which is passed scurely to research staff at the university. The research team then take responsibility for contacting service users and inviting them to participate. Table 1 below provides further detail on the recruitment procedure. SARC service users may also self-refer into the study. Anyone aged 18 years or over who has visited a SARC in England since 1st September 2019 is eligible for enrolment via direct contact with the research team. Details about how to enrol are available on the study website.

Number of participants required

At each of the 15 participating SARCs and via some self-referral, a total of 2500 individuals aged ≥18 years are being considered for eligibility. Based on previous studies [21 22] our pilot work, and recruitment and retention to date we estimate an enrolment rate of 13.5% (338 participants) but with fairly low attrition at 20% by 12 month follow-up, leaving approximately 270 individuals' data for the analysis at that time (see estimated flow of participants in Figure 1).

With data across baseline and two follow-up timepoints (6 and 12 months), a maximum of four levels of variability across SARC models of care (e.g. SARC service leadership model, integration of ISVA service) and up to ten covariates (e.g. time since index assault that led to SARC access, type of index assault), this sample-size will mean we have 90% power with alpha error probability held at 0.05 to detect a small-to-moderate effect (Cohen's f).

Figure 1 about here

Data collection

The form used by SARC staff to screen service users for the research generates data on gender; age; ethnicity; offence characteristics; referral to SARC; and indicators of vulnerability. At baseline, we collect additional socio-demographic information on marital/relationship status; nationality; immigration status; number of children; sexual identity; disability; religion; education; employment status and income; and accommodation. Baseline data is gathered by structured interviews, by telephone, video link or in-person by trained research staff who enter responses directly into secure bespoke study software

For peer review only - http://bmjopen.bmj.com/site/about/guidelines.xhtml

Table 1. Procedure for involving adult service users in the MESARCH cohort study

1. *user receives service from SARC staff.*

2. *SARC staff member screens service user for eligibility. All eligibility information is anonymous and conveyed to the project team using secure sharing platform.*

3. *Eligible service users are briefly informed about the study at an appropriate point at the SARC and a referral is made to third/voluntary sector. A person may be invited to provide consent to be contacted (Level 1 consent) at the SARC in particular if they decline or are not eligible for a referral to an ISVA.*

4. *Eligible service users are approached mainly by Independent Sexual Violence Advisors (ISVA) who provide brief explanation about the project and officially invites service user to consider being involved in the study. Recruiting SARC service users through the third sector requires the SARC to pass on the service user's unique project ID in order to enable the recruitment process to continue beyond the SARC setting. Next, consent is requested to pass contact details to the project team ('Level 1' consent completed).*

5. *Additional approaches for enabling recruitment include (i) having members of the project team ready to speak by phone or video link to a service user if the SARC worker/ISVA believes this is an appropriate approach for a particular service user; (ii) showing a short video message of invitation co-produced with our Lived Experiences Group which could be used by SARC staff/ISVA to explain about the project (available on our project website and accessible using a QR code on study brochure); (iii) opening study up to self-referrals by survivors who have used SARCs in England during the inclusion period.*

6. *Once the contact information has been passed on, there is no more involvement of the SARC/third sector agency except in circumstances such as the participant requires support and has agreed for us to notify the SARC/third sector agency.*

7. *A trained project team member makes contact within 1 week of receiving the research referral consent form from the SARC or third sector worker, and contact is made in line with service user preferences for example by email, text or phone call.*

8. *Once contact is made by the project team at baseline, the project team will follow recruitment and safety protocols, explain study purpose and gain full consent ('Level 2' consent).*

9. *When consent to take part in the study is established (using email or text or signed consent form), baseline data are collected using a range of options. These include offering a structured telephone interview, a weblink to complete the data collection online, an interview in person or via Skype (or alternative). Those submitting baseline data will be considered 'enrolled' in MESARCH.*

10. *Follow-up is undertaken according to the service users' preferences at 6 and 12 months consistent with our previous work* [23]

linked to Qualtrics survey software. We enquire about adverse childhood experiences using the WHO ACE International Questionnaire and screen for past year domestic violence using

For peer review only - http://bmjopen.bmj.com/site/about/guidelines.xhtml

the ACTS [24] to enable us to describe the sample and conduct sub-group analyses. All other

Outcome	Measure*	Timepoint
PTS	PCL-5	Baseline, 6 months, 12 months
Depression	CESD-R-10	Baseline, 6 months, 12 months
Quality of life	WHOQoL-Bref	Baseline, 6 months, 12 months
Eating issues	SCOFF<br>BEDS-7	Baseline, 6 months, 12 months
Health related quality of life	EQ5D-5L	Baseline, 6 months, 12 months
Capability	ICEpop CAPability	Baseline, 6 months, 12 months

baseline measures focus on standardised timeframes according to the measure, or the period since participants attended the SARC. Follow-up is taking place on two occasions over the course of the project, at 6 months and 12 months. Our proposed methods for retaining participants are informed by a number of large studies of violence and health [25] including our own previous work in the health field [26]. These include gathering a range of contact details from participants at baseline - safe telephone numbers, postal/email addresses; use of reminders; and providing a small incentive (i.e. £10 following baseline completion, £10 at 6 months and £10 at 12 months).

Outcomes

Table 2 shows the measures at each time point. The primary outcome is post-traumatic stress (PTS), widely endorsed in the literature [4 9] and through our scoping work with individuals with lived experience and service providers, as a primary health issue for survivors and an absence of diagnosis, or reduction in symptoms may mark improvement or recovery in a person who has experienced sexual violence. We have developed a set of items to assess sexual health, including STI diagnosis, condom use; pregnancy and pregnancy outcomes, and reproductive coercion [27 28]. We are screening for sexual violence re-exposure at follow-up. This is not assessed at baseline beyond the items reported in the SARC screening form, and, in line with UK pre-trial therapy guidelines, discussion of the experience of sexual violence that led the person to seek help at the SARC is not encouraged in order to protect victims' testimonies. Our approach to measuring health and other costs using the ICECAP-A [29] and EQ5D-5L [30] is outlined in *Economic Evaluation* below. We have also developed a comprehensive evaluation of SARC service users onwards service utilisation and satisfaction with care that considers the many sectors that are implicated in meeting the needs of survivors in the aftermath of an assault or abuse.

For peer review only - http://bmjopen.bmj.com/site/about/guidelines.xhtml

	measure for Adults (ICECAP-A)	
Sexual health	Bespoke measure	Baseline, 6 months, 12 months
Alcohol use	AUDIT-C	Baseline, 6 months, 12 months
Drug use	DUDIT	Baseline, 6 months, 12 months
Resource use	Bespoke measure	Baseline, 6 months, 12 months
Adverse Childhood Experience	WHO ACEs	Baseline
Intimate Partner Violence	ACTS 4 item screen<br>CAS	Baseline<br>6 months, 12 months
Sexual violence	Bespoke measure	6 months, 12 months
Suicidality and self-harm	APMS	Baseline, 6 months, 12 months

Table 2. Outcome measures and the timepoint at which they are collected.

*In light of the COVID-19 pandemic and the social distancing measures that were necessitated by this at the end of March 2020, our interviews going forward with participants at baseline, 6 months and 12 months will include an additional open-ended question in order to capture what impact COVID-19 this has had for individuals. The following question will be asked "We would like to understand if and how Covid-19 has affected your capacity to look after yourself in the aftermath of your experience of sexual assault and get the care and support you need. Please share with us any views you have on this." To understand how people's their lives have changed as a result of the sexual assault (for example, social lives, work, service use), it is important that we ask this question early on to frame the remainder of the interview and help separate the impacts from Covid-19 related measures.

Data analysis

Analysis of covariance (ANCOVA), powered to detect fixed effects, main effects, and interactions will be conducted. We will then carry out multinomial logistic regression. Based on the sample size calculation, sensitivity power analysis indicates we would be sufficiently powered to detect odds ratios >1.25 and <0.8, equitable to identifying a small effect size (Cohen's *d* (>0.2). Using exploratory path analysis, structural relationships between the observed variables will be modelled to explore potential sequential relationships between such variables, and the total, indirect, and direct effects will be computed accordingly.

Exploratory subgroup analyses will assess how trajectories compare for different subgroups of survivors attending SARCs through assessment of those minority groups large enough to enable meaningful statistical comparison. Evidence of interaction for type of service according to demographic subgroup will be regarded as statistically significant if p-values of 0.01 or smaller are found. We aim to compare outcomes for different gender and sexual identities; ethnicities; levels of disability; chronic mental health problems.

For peer review only - http://bmjopen.bmj.com/site/about/guidelines.xhtml

We will also examine if PTS trajectories are influenced by other organisational aspects such as size of centre, location, interventions/services available, and length of wait to access services. The final subgroup analysis will examine the effect of setting of post-crisis counselling.

Economic evaluation

Design

The economic analysis will compare the costs and outcomes associated with different organisational aspects of SARC service delivery. If some SARC models are more strongly associated with reducing PTS and improving quality of life and other mental, physical and sexual health outcomes than others, there are likely to be important cost implications for the healthcare sector, for the wider public sector, and for society as a whole.

Data collection

Resource use data will be collected prospectively to estimate the costs associated with different models of SARC service delivery. The resource use to be monitored will include: 1) the cost of service use within SARCs (e.g. consultations, treatment etc.); 2) NHS and other public sector resource use after initial attendance at SARCs (e.g. GP visits, sexual health visits); 3) costs associated with the treatment of PTS and other relevant conditions; 4) wider public sector resource use, for example in relation to social care, housing, and other social welfare systems; 5) costs experienced by service users and family members. Information on unit costs or prices will be sourced to attach to each resource use item (e.g. [31]). Health-related quality of life data will be collected using the EQ5D-5L instrument which is widely used for those with PTSD and related conditions [32].

Economic analysis

In order to compare the costs and benefits of different SARC service delivery models, both a within study analysis and a model based economic analysis will be undertaken. The main economic analysis will assess cost-effectiveness based on incremental cost per quality-adjusted life year (QALY) gained at 6 and 12 months, with a secondary analysis of cost per case of PTS avoided at 12 months. If the results of the cohort study show that there are significant differences in the effectiveness of SARC delivery models, in terms of reducing PTS and improving other health outcomes, it will be necessary to assess the cost-effectiveness of the SARC delivery models in the longer term, to take into account the

For peer review only - http://bmjopen.bmj.com/site/about/guidelines.xhtml

impact on an individual's quality of life and productivity. Therefore, if deemed necessary, based on the results of the cohort study, we will use a decision-analytic model to evaluate the longer-term impacts of the different types of service delivery (for up to five years, if data allow). The model development process will use, as a starting point, other models developed for PTS and related conditions (e.g. [33]). We will use both deterministic and probabilistic sensitivity analyses to explore the effects of inherent uncertainty in the estimates on the results [34]. For the longer-term analyses, discounting will be undertaken to reflect recommendations by NICE [35].

Pilot study

A pilot study was conducted with baseline recruitment over three months between April and June 2019, at one SARC site. The purpose was to examine the feasibility of the methods for the main cohort study and identify required design modifications. We assessed the feasibility of our approach to inviting participants into the study, data gathering procedures, and retention (e.g. rates and use of incentives). At the SARC, we discussed the project with staff members, built awareness about the rationale, explained inclusion/exclusion criteria and use of the screening form to record eligibility, and agreed on the most appropriate staff members to approach potential participants at different stages (as one feature of the research delivery that will vary from one SARC to another). Once set-up was complete, all eligible service users over a 3-month period were invited into the pilot project by SARC workers. The pilot included telephone interviews at baseline and one follow-up only, at 3 months. Data collection included a set of questions about the experience of being invited into and participating in the study.

Over three months, 43 service users attended the pilot SARC (classed as small relative to other English SARCs). Staff tended to be highly conservative in applying the eligibility criteria, voicing concerns about mental ill-health and overburdening the service users (n=15). Of the 28 service users identified as eligible, 11 lost contact with the SARC prior to being invited into the study and three declined to have their contact details passed on. Of the 14 people who agreed to be contacted by the research team, four could not be contacted and ten were successfully enrolled. From those ten participants, there were no missing data and retention rates at 3 months were 100%. We encouraged think aloud responses to items and requested feedback about people's experiences of the pilot. These were all positive comments. For example, *"I really enjoyed being a part of the study. It felt great to be able to take an unfortunate event and turn it into something positive and it's nice knowing that what I contributed will go on to help others" (23yr old female participant in pilot study*).

For peer review only - http://bmjopen.bmj.com/site/about/guidelines.xhtml

For the main study, we are providing clearer guidance to staff about the exceptional circumstances under which a person should be identified as ineligible. We are aware of the circumstances of people's lives that make research participation difficult, but we believe they should, wherever possible, be given full choice around being involved or not. Thus, a reduced rate of exclusion is anticipated. This is continuously monitored during recruitment and further intervention implemented accordingly when deemed necessary. We have continued to focus our efforts on minimising the numbers of individuals who are potentially 'lost' prior to being invited into the study. Not all service users take up the offer of an ISVA, which has been our primary avenue to reach potential participants through a targeted strategy of training staff and promoting structured follow-up of all new clients who are referred from SARCs. Thus, in addition to working with ISVAs, have cascaded information across the voluntary sector and sexual health services to enable self-referral. Our systems are set up to enable us to link self-referrals to original SARC index visits with screening forms once consent is provided. We are aware from another similar study [21 22] our own pilot study and our early recruitment experiences of the challenges in recruitment. Issues have included staff not wanting to mention the study or invite participation, or simply finding it difficult to include it in engagement they have with survivors. We have worked hard to gain buy-in across services to try to mitigate this and have found that persistence and good communication with services is key, particularly during the Covid-19 pandemic which has caused much disruption to service provision. Loss of contact with survivors by services and service users not wanting to engage has been a challenge. We oversampled SARC sites however (targeting 15 when 12 may have got us to the required target), and our methods appear to achieve good retention rates, with low levels of missing data based on pilot study findings and early recruitment experiences.

For peer review only

Qualitative study

Design

There will be a nested qualitative, interview-based study to enable a greater depth of understanding of the factors associated with better outcomes for survivors of sexual violence participating in the adult cohort study. It will include a community-based comparison sample. We will include around 50 individuals aged 18 years and above.

For peer review only - http://bmjopen.bmj.com/site/about/guidelines.xhtml

The study will employ narrative methods [36] as these provide participants with the opportunity to give their accounts of a particular experience, free of the assumptions of the interviewer or research team and empowering them to structure the stories of what happened, and their meanings as understood by them. Narrative methods have been applied extensively in explorations of experiences relevant to SV and rape (e.g. [37-39] and lend themselves well to gaining insight into the ways in which people get to grips with potentially devastating disruptions to their everyday lives (e.g. [40]).

Recruitment of participants

Participants who have participated in the adult cohort study will be recruited in order to gain insight into factors that were experienced positively and negatively from a range of narratives. This will provide the project team with rich data about experiences outside of SARC models of care that were valued, as well as those within SARC models. In collaboration with our Lived Experiences Group and professionals who support survivors, we will devise a sampling framework and detailed recruitment strategy that is sensitive to the needs of, and acceptable to, the target population. We propose that this will involve recruiting adults from the SARC cohort study after their 12-month measures are collected. We will aim to recruit around 30 cohort members in total and apply maximum variation sampling in order to over-represent service users whose voices are not typically included in this type of research (e.g. people affected by sex work, members of BAME and LBGTQ+ communities).

Recruitment of non-SARC service users

We will also seek to recruit people via third sector partners and stakeholders who support survivors of SV. This will ensure wider experiences are included in the data (approximately 20 individuals) and will help the research team to better understand barriers to SARC access among those offered a referral by other services but who have not used SARCs.

Procedure

Participants who express an interest in being interviewed will be given time and support to prepare for the narrative interview having had a full explanation of what this involves and ensuring they are comfortable to provide their story about experience of services following SV. Participants will be offered the choice of a face-to-face (where Covid restrictions allow), online video conference, or telephone interview at a time and location convenient to them

For peer review only - http://bmjopen.bmj.com/site/about/guidelines.xhtml

and either with or without the support of a person of their choosing. The interview guide will be developed in partnership with survivors of SV and those who support them within our lived experience group. It is likely to include an opportunity for the participants to talk generally about themselves and how they are, and to discuss any concerns they may have about the narrative interviews they are engaging with. The interviewer will discuss any concerns and ensure that the participant is happy to talk about their recovery journey. The interviewer will likely suggest the participant starts at an appropriate moment in discussions by saying that he/she/they are, 'interested in finding out about your experiences of accessing help or support and the impact on your health and wellbeing, after experiencing sexual violence' and, 'you can begin to tell us your experiences of receiving support and your recovery journey so far, at the point where you first sought help in relation to your experience of sexual assault, or wherever it feels right to start'. We will be clear through our participant information sheet and consent procedures that we are not asking participants to talk about the incident(s) that has/ve led them to seek help and support. Interviews will be audio-recorded with participants' permission and transcribed verbatim.

Analysis of qualitative data

Data will then be subject to narrative analysis [36] to draw out the meanings ascribed to participants' experiences and to identify both unique elements and commonality or themes across experiences. This process will be led by KB, with support from GC and another research assistant. Narrative analysis is divided into two distinct phases. The first is a descriptive phase which, following thorough reading and familiarisation with the transcripts, involves devising summaries of each narrative to pull out the key features and identify sub-plots as well as overarching story arcs. Similarity between different narrative summaries, as well as key differences, will also be identified at this stage to form the basis of a coding frame. In the second stage, a range of theoretical perspectives will be considered in order to interpret and make sense of the narratives and the coding frame. To achieve this we will work collaboratively with co-investigators and Lived Experience Group members, considering the range of options and the ways in which they may or may not aid interpretation of the data. This process will be used to support interpretation of quantitative findings from the cohort data.

ETHICS AND DISSEMINATION

The pilot study and main cohort study have received ethical approval from Coventry University (pilot: P76598; main study: P86669) and NHS Research Ethics (pilot:

For peer review only - http://bmjopen.bmj.com/site/about/guidelines.xhtml

18/WM/0376; main study: 19/EM/0198). NHS Health Research Authority approval was also provided before data collection began.

The research is supported by an independent study steering committee (SSC), a data monitoring and ethics committee (DMEC) and a Patient and Public Involvement (PPI) group, known as the Lived Experiences Group (LEG). Every aspect of the research to date has involved input from the LEG, including the research design at the funding application stage and the subsequent detailed consideration of operationalising the protocol since funding was awarded. Members of the LEG are also involved in the SSC to offer their support to the independent considerations of this committee. The SSC have been in place since study set up and are made up of a range of relevant academic, government, non-government and independent agencies and experts who can offer guidance and support on the challenges of conducting the research throughout. One example of SSC and LEG input includes working with them to refine our choice of measures, in particular, appropriate ways to assess sexual violence exposure in this high-risk population [41]. A further example is having worked with some of our LEG members on the development of short videos to introduce the study to professionals and survivors to support recruitment. Both of these films can be accessed on the study website at http://mesarch.coventry.ac.uk/getting-involved/. The DMEC is meeting three times throughout the cohort study (baseline, post 6 months FU and post 12 months FU) to assess data, analyses, and whether any evidence of harm should lead to a recommendation that the study ceases.

The experience, safety and wellbeing of survivors, participants and staff is of paramount importance in this research. All staff involved in delivering this cohort study and the wider research activities in the funded project have undergone specialist training from two voluntary sector organisations who work to support survivors of sexual violence. We have developed a detailed safety protocol to define clearly what the actions of staff should be if there are any safety or wellbeing concerns raised during research procedures. These are with regards to potential participants, participants and any children or other dependents, and staff. A central guiding principle of the research is that being involved as a participant should feel like the opposite of being a victim of sexual violence. In other words, it should involve feeling empowered, respected, and in control at all times. In fact, in our experience of data collection to date, during the pilot and the first participants at baseline in the main cohort study, people report exactly that. They are happy to and want to participate in the research because it is an opportunity for them to pro-actively contribute something positive to improve services in the future. It is their decision to do so and they are in control of the answers and information they provide.

For peer review only - http://bmjopen.bmj.com/site/about/guidelines.xhtml

One issue that it has been particularly pertinent to consider is the possibility that data provided by participants pursuing a prosecution may be subject at any time to subpoena by the courts. Participants are made aware of this before providing consent, and the focus of the research is not what happened during any alleged incident of sexual violence. To minimise the likelihood that we will collect data relating to any incidences of sexual violence, our safety protocol involves a requirement that staff remind participants about the possibility of subpoena if ever they begin to disclose information that goes beyond answers to the health and wellbeing measures and experience of service use.

With regards to the safety and wellbeing of our staff, we hold a weekly project meeting where issues can be raised, and staff are encouraged to talk to a senior colleague within the team if at any time they are experiencing difficulties with the nature of the study and the data they are handling. Telephone counselling is available 24/7 to all staff and if needed bespoke support will be arranged with our expert rape crisis support stakeholders and partners in the research.

Dissemination of the findings will be supported by our SSC and the LEG through a range of channels in the future. In addition to standard publication in peer reviewed academic journals and at academic conferences, we will present findings at policy and practice-based conferences nationally and internationally. We will publish findings in lay language on our study website and produce other forms of lay publications targeted at all agencies and organisations with a remit for addressing sexual violence and its consequences in society. We will work with NHS England who commissioned the research to ensure the findings are translated appropriately into policy and practice.

Author contributions: LOD conceived the study design with support from GF, RM, RC, LJ and KB. LOD led the successful application for funding. GC and AM are members of the project delivery team responsible for recruitment and data collection. KB drafted the manuscript based on the approved study protocol V3.3 and all co-authors read, contributed to and commented on drafts.

FUNDING STATEMENT

This work is supported by the National Institute for Health Research (Health Services & Delivery Research programme) grant number 16/117/04. The views expressed are those of the author(s) and not necessarily those of the NIHR or the Department of Health and Social Care.

For peer review only - http://bmjopen.bmj.com/site/about/guidelines.xhtml

COMPETING INTERESTS STATEMENT

The authors have no competing interests to declare

ACKNOWLEDGEMENTS

We acknowledge the contributions of Millie Gant, Juniper Lodge SARC; Dianne Whitfield, Rape Crisis England, Priya Tek-Kelsi, the project PPI co-ordinator for her significant contribution to getting this work off the ground; and all the members of our lived experience group, the study steering committee and the data monitoring and ethics committee for all their contributions and support.

Word count: 5032 words

For peer review only

For peer review only - http://bmjopen.bmj.com/site/about/guidelines.xhtml

References

1. ONS. Violent crime and sexual offences-Intimate personal violence and serious sexual assault., 2015.
2. Krug EG, Mercy JA, Dahlberg LL, et al. The world report on violence and health. *The lancet* 2002;360(9339):1083-88.
3. ONS. Sexual offending: victimisation and the path through the criminal justice system, 2018.
4. World Health Organization. Global and Regional Estimates of Violence Against Women: Prevalence and Health Effects of Intimate Partner Violence and Non-Partner Sexual Violence. Geneva (CH): World Health Organization 2013.
5. Campbell JC. Health consequences of intimate partner violence. *The lancet* 2002;359(9314):1331-36.
6. Tewksbury R. Physical, Mental and Sexual Consequences. *International Journal of Men's Health* 2007;6(1):22-35.
7. Coxell AW, King MB. Male victims of rape and sexual abuse. *Sexual and Marital Therapy* 1996;11(3):297-308.
8. Walker J, Archer J, Davies M. Effects of rape on men: A descriptive analysis. *Archives of sexual behavior* 2005;34(1):69-80.
9. Pietrzak RH, Goldstein RB, Southwick SM, et al. Prevalence and Axis I comorbidity of full and partial posttraumatic stress disorder in the United States: results from Wave 2 of the National Epidemiologic Survey on Alcohol and Related Conditions. *Journal of anxiety disorders* 2011;25(3):456-65.
10. Liu H, Petukhova MV, Sampson NA, et al. Association of DSM-IV posttraumatic stress disorder with traumatic experience type and history in the World Health Organization World Mental Health Surveys. *JAMA psychiatry* 2017;74(3):270-81.
11. Guina J, Nahhas RW, Kawalec K, et al. Are gender differences in DSM-5 PTSD symptomatology explained by sexual trauma? *Journal of interpersonal violence* 2019;34(21-22):4713-40.
12. Dubourg R, Hamed J, Thorns J. The economic and social costs of crime against individuals and households 2003/04. *Home Office online report* 2005;30(05)
13. Organization WH. Guidelines for medico-legal care of victims of sexual violence. 2003
14. England N. Public Health Functions to be Exercised by NHS England–Service Specification: Sexual Assault Referral Centres. *NHS England* 2018
15. England N. Sexual Assault and Abuse no date [Available from: https://www.england.nhs.uk/commissioning/health-just/sexual-assault-and-abuse/#sarcs accessed 09/03/21 2021.
16. Lovett J, Regan L, Kelly L. Sexual Assault Referral Centre s : developing good practice and maximising potentials2004.
17. Weare S, Hulley J, Pattinson B. A Review of the National Male Survivors Helpline and Online Service-Project Report: Lancaster University, 2019.
18. Basile KC, Breiding MJ, Smith SG. Disability and risk of recent sexual violence in the United States. *American journal of public health* 2016;106(5):928-33.
19. Ellsberg M, Arango DJ, Morton M, et al. Prevention of violence against women and girls: what does the evidence say? *The Lancet* 2015;385(9977):1555-66.
20. O'Driscoll C, Flanagan E. Sexual problems and post-traumatic stress disorder following sexual trauma: A meta-analytic review. *Psychology and Psychotherapy: Theory, Research and Practice* 2016;89(3):351-67.
21. Abrahams N, Seedat S, Lombard C, et al. Study protocol for a longitudinal study evaluating the impact of rape on women's health and their use of health services in South Africa. *BMJ open* 2017;7(9)

For peer review only - http://bmjopen.bmj.com/site/about/guidelines.xhtml

22. Abrahams N, Mhlongo S, Chirwa E, et al. Rape survivors in South Africa: analysis of the baseline socio-demographic and health characteristics of a rape cohort. *Global Health Action* 2020;13(1):1834769. doi: 10.1080/16549716.2020.1834769
23. Hegarty KL, Gunn JM, O'Doherty LJ, et al. Women's evaluation of abuse and violence care in general practice: a cluster randomised controlled trial (weave). *BMC Public Health* 2010;10(1):1-11.
24. Hegarty K, Spangaro J, Kyei-Onanjiri M, et al. Validity of the ACTS Intimate Partner Violence Screen in antenatal care: A cross sectional study. *BMC Public Health* under review 2021 doi: 10.21203/rs.3.rs-264487/v1
25. Ford-Gilboe M, Varcoe C, Scott-Storey K, et al. A tailored online safety and health intervention for women experiencing intimate partner violence: the iCAN Plan 4 Safety randomized controlled trial protocol. *BMC Public Health* 2017;17(1):1-12.
26. Hegarty K, O'Doherty L, Taft A, et al. Screening and counselling in the primary care setting for women who have experienced intimate partner violence (WEAVE): a cluster randomised controlled trial. *The Lancet* 2013;382(9888):249-58. doi: https://doi.org/10.1016/S0140-6736(13)60052-5
27. Grace KT, Anderson JC. Reproductive Coercion: A Systematic Review. 2018;19(4):371-90. doi: 10.1177/1524838016663935
28. Maxwell L, Devries K, Zionts D, et al. Estimating the effect of intimate partner violence on women's use of contraception: a systematic review and meta-analysis. *PLOS ONE* 2015;10(2):e0118234.
29. Al-Janabi H, Flynn TN, Coast J. Development of a self-report measure of capability wellbeing for adults: the ICECAP-A. *Quality of life research* 2012;21(1):167-76.
30. EQ5D ERF. EQ-5D-5L [Available from: https://euroqol.org/eq-5d-instruments/eq-5d-5l-about/ accessed 1st September 2019.
31. Curtis L, Burns A. Unit Costs of Health and Social Care. Canterbury: Personal Social Services Research Unit, University of Kent 2015.
32. Ophuis RH, Lokkerbol J, Heemskerk SC, et al. Cost-effectiveness of interventions for treating anxiety disorders: A systematic review. *Journal of affective disorders* 2017;210:1-13.
33. Mihalopoulos C, Magnus A, Lal A, et al. Is implementation of the 2013 Australian treatment guidelines for posttraumatic stress disorder cost-effective compared to current practice? A cost-utility analysis using QALYs and DALYs. *Australian & New Zealand Journal of Psychiatry* 2015;49(4):360-76.
34. Briggs AH, Weinstein MC, Fenwick EA, et al. Model parameter estimation and uncertainty: a report of the ISPOR-SMDM Modeling Good Research Practices Task Force-6. *Value in Health* 2012;15(6):835-42.
35. Woolf SH, Purnell JQ, Simon SM, et al. Translating Evidence into Population Health Improvement: Strategies and Barriers. *Annual Review of Public Health* 2015;36(1):463-82. doi: 10.1146/annurev-publhealth-082214-110901
36. Murray M. Narrative Psychology in Qualitative Psychology In: Smith JA, ed. Qualitative psychology: A practical guide to research methods: Sage Publications, Inc 2003.
37. Anderson S, Jia JX, Liu V, et al. Violence prevention and municipal licensing of indoor sex work venues in the Greater Vancouver Area: narratives of migrant sex workers, managers and business owners. *Culture, health & sexuality* 2015;17(7):825-41.
38. Kim T, Draucker CB, Bradway C, et al. Somos Hermanas Del Mismo Dolor (We are sisters of the same pain) Intimate partner sexual violence narratives among Mexican immigrant women in the United States. *Violence against women* 2017;23(5):623-42.
39. Foster JM, Hagedorn WB. Through the eyes of the wounded: A narrative analysis of children's sexual abuse experiences and recovery process. *Journal of child sexual abuse* 2014;23(5):538-57.

For peer review only - http://bmjopen.bmj.com/site/about/guidelines.xhtml

40. Becker G. Disrupted lives: How people create meaning in a chaotic world: Univ of California Press 1997.
41. Probst DR, Turchik JA, Zimak EH, et al. Assessment of sexual assault in clinical practice: Available screening tools for use with different adult populations. *Journal of Aggression, Maltreatment & Trauma* 2011;20(2):199-226.

For peer review only

For peer review only - http://bmjopen.bmj.com/site/about/guidelines.xhtml

Figure 1. Flowchart of service users aged 18 years and above in the MESARCH cohort study

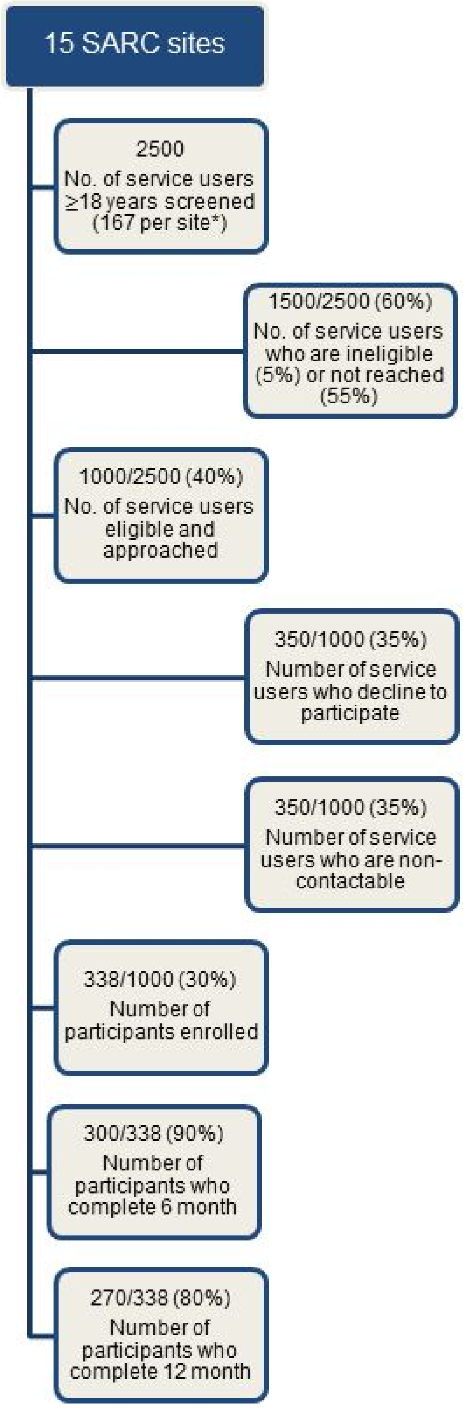

BMJ Open

Multi-disciplinary Evaluation of Sexual Assault Referral Centres (SARCs) for better Health (MESARCH): Protocol for a one-year cohort study examining health, wellbeing and cost outcomes in adult survivors of sexual assault attending SARCs in England

Journal:	*BMJ Open*
Manuscript ID	bmjopen-2021-057449.R1
Article Type:	Protocol
Date Submitted by the Author:	24-Jan-2022

Complete List of Authors:	O'Doherty, Lorna; Coventry University, Faculty of Health and Life Sciences<br>Carter, Grace; Coventry University<br>Lutman-White, Eleanor; Coventry University, Institute for Health and Wellbeing<br>Caswell, Rachel; University Hospitals Birmingham NHS Foundation Trust, Rachel Caswell<br>Jackson, Louise; University of Birmingham, Health Economics Unit<br>Feder, Gene; University of Bristol, Community based medicine<br>Heron, Jon; University of Bristol, Centre for Academic Mental Health,<br>Morris, Richard; University of Bristol, School of Social & Community Medicine<br>Brown, Katherine; University of Hertfordshire, Department of Psychology, Sports Science and Geography

**Primary Subject Heading**:	Health services research
Secondary Subject Heading:	Public health, Global health, Health economics, Sexual health, Mental health
Keywords:	SEXUAL MEDICINE, PUBLIC HEALTH, MENTAL HEALTH, HEALTH SERVICES ADMINISTRATION & MANAGEMENT

SCHOLARONE™
Manuscripts

Multi-disciplinary Evaluation of Sexual Assault Referral Centres (SARCs) for better Health (MESARCH): Protocol for a one-year cohort study examining health, wellbeing and cost outcomes in adult survivors of sexual assault attending SARCs in England

Lorna O'Doherty[1], Grace Carter[1], Eleanor Lutman-White[1], Rachel Caswell[2], Louise Jackson[3], Gene Feder[4], Jon Heron[5], Richard Morris[4], & Katherine E Brown[6]

1. Institute for Health and Wellbeing, Coventry University
2. Department of Sexual Health and HIV Medicine, University Hospitals Birmingham,
3. Health Economics Unit, Institute of Applied Health Research, University of Birmingham
4. Centre for Academic Primary Care, Population Health Sciences, Bristol Medical School, University of Bristol
5. Centre for Academic Mental Health, Population Health Sciences, Bristol Medical School, University of Bristol
6. Department of Psychology, Sports Science and Geography, School of Life and Medical Sciences, University of Hertfordshire

Corresponding author: Professor Katherine Brown

Department of Psychology, Sports Science and Geography, School of Life and Medical Sciences, University of Hertfordshire, Hatfield, AL10 9AB

Email: k.brown25@herts.ac.uk

Word count: 5747

For peer review only

ABSTRACT

Introduction

Sexual violence is commonplace and has serious adverse consequences for physical and mental health. Sexual Assault Referral Centres (SARCs) are viewed as a best practice response. Little is known about their effectiveness and cost-effectiveness. Long-term data on the health and wellbeing of those who have experienced rape and sexual assault are also lacking.

Methods and analysis This is a mixed-methods protocol for a one-year cohort study aiming to examine health and wellbeing in survivors of sexual violence after attending a SARC in England. Quantitative measures are being taken at baseline, 6, and 12 months. Post-traumatic stress (PTS) is the primary outcome (target N=270 at 12-month follow-up). Secondary measures include anxiety, depression, substance use and sexual health and wellbeing. Using mixed-effects regression, our main analysis will examine whether variation in SARC service delivery and subsequent mental health care is associated with improvement in trauma symptoms after12 months. An economic analysis will compare costs and outcomes associated with different organisational aspects of SARC service delivery and levels of satisfaction with care. A nested qualitative study will employ Narrative Analysis of transcribed interviews with 30 cohort participants and 20 survivors who have not experienced SARC services.

Ethics and dissemination

The research is supported by an independent study steering committee (SSC), data monitoring and ethics committee (DMEC) and Patient and Public Involvement (PPI) group. A central guiding principle of the research is that being involved should feel like the opposite of being a victim of sexual violence; and be experienced as empowering and supportive. Our PPI representatives are instrumental in this, and our wider stakeholders encourage us consider the health and wellbeing of all involved. We will disseminate widely through peer-reviewed articles and non-academic channels to maximise the impact of findings on commissioning of services and support for survivors.

ISRCTN registry: https://doi.org/10.1186/ISRCTN30846825

For peer review only - http://bmjopen.bmj.com/site/about/guidelines.xhtml

ARTICLE SUMMARY

Strengths and limitations of this study

- The study proposes the most substantial investigation to date in the UK of health, wellbeing and service utilisation in survivors of sexual assault and rape
- The study applies a prospective, longitudinal design with a national sample of adults in England taking up SARC services following experience of sexual assault or rape, including all genders, to look at outcomes over one year
- The study considers a wider definition of sexual health than just STI acquisition
- The study uses innovative approaches to recruitment that address barriers to research participation in the target population, including challenges created by Covid-19 and is supported by a wide range of stakeholders nationally including a group of people with lived experience of sexual abuse
- Relatively few survivors of sexual assault and rape have used SARCs, so generalisability to all people who have experienced sexual violence and abuse may be limited

For peer review only

INTRODUCTION

Sexual violence is commonplace, with evidence suggesting that one in five women and one in twenty-five men in England and Wales have experienced sexual assault since the age of 16[1]. Sexual violence refers to any sexual act, or attempt at a sexual act, or an act directed at a person's sexuality, involving coercion[2]. Sexual violence includes but is not restricted to: rape, sexual assault, child sexual abuse, sexual harassment, rape within marriage and relationships, forced marriage, so-called honour-based violence, female genital mutilation, trafficking, sexual exploitation, and ritual abuse[2]. Sexual violence often goes unreported to the police, though recent shifts in the public understanding of sexual violence and developments that include the *#metoo* movement on social media have likely contributed to year-on-year increased rates of reporting[3]. In the year to March 2017, one in six people in England and Wales reported their experience to the police - 121,187 incidents of sexual assault in total, representing a 14% increase on the previous year[3].

The serious and devastating effects of sexual violence are a well-documented public health burden[4]. They include a range of immediate and long-term physical and mental health consequences. For women who have experienced sexual violence, physical health consequences include unwanted pregnancy, sexually transmitted infections (STIs)[4], urinary tract infections, painful sex, chronic pelvic pain and vaginal bleeding[5]. For male victims, physical health consequences include genital and rectal injuries and erectile dysfunction[6]. The mental health sequelae of sexual violence have been found to be equally substantial across different population groups[4 6-8]. Post-traumatic stress (PTS) is common amongst those who have experienced sexual violence,[9 10] with incidence and severity being similar in men and women[11]. Other mental health consequences include alcohol use disorders, eating disorders, anxiety, depression, self-harm and suicidality[4]. In addition, experiencing mental health problems such as lifetime PTS is associated with increased risk of other long-term health conditions including hypertension, cardiovascular disease and gastrointestinal problems[9]. Thus, sexual violence produces significant health burden, with each experience of rape in adults estimated to cost over £73,000 in psychological and physical impacts of injuries/illnesses and resultant health service use alone[12]. The wider economic costs to society reach over £8billion per year[12].

Given the substantial personal, societal and economic costs of sexual violence, an effective and consistent response for survivors is imperative. In the 2003 guidelines on the provision of care for victims of sexual violence, the World Health Organization recommended that an initial response to survivors should include medico-legal and health services provided at the same time, in the same location and preferably by the same health practitioner. Policy-

For peer review only - http://bmjopen.bmj.com/site/about/guidelines.xhtml

makers, commissioners and health workers have been encouraged to develop this model of service provision[13]. In the USA for example, many states offer Sexual Assault Nurse Examiner (SANE) programs or Sexual Assault Response Teams (SARTs) to provide these recommended services. In England and other parts of the United Kingdom, there has been a steady growth in Sexual Assault Referral Centres (SARCs) as a best practice response for survivors after incidents of sexual assault or rape[14]. SARCs, as a form of medico-legal and health service, are intended to co-ordinate all of the care and support needs for survivors. This may include immediate post-incident crisis support, forensic medical examination (FME) for the purposes of collecting evidence needed to prosecute perpetrators, referral to sexual health and other healthcare services, referral for mental health care needs (e.g. counselling and therapy) and the provision of an independent sexual violence advisor (ISVA) for ongoing support, particularly if that includes dealing with the criminal justice system. At the time of writing, there are reportedly 47 SARCs in England including a number of specialist paediatric sites[15], operating with considerable variation in their service models[16]. Currently, little is known about the effectiveness and cost-effectiveness that the different service models may represent in addressing the physical and mental health outcomes for survivors of sexual violence outlined above. In addition, although the substantial negative impacts of sexual violence are well-documented[4] much less is known about the longer-term health and wellbeing outcomes for survivors. One exception is a recent study in South Africa which followed up female rape survivors over three years and, controlling for confounding variables, has shown they had a 60% increased risk of contracting HIV in that time compared to control group women who had not been raped[17-19]. Data related to models of care survivors receive and health outcomes remain limited, however. Similarly, there is a paucity of data on (mental) health care access after SARC and understanding of how different service user characteristics might moderate the benefits of attending SARC (and other specialist services for sexual assault and rape), in particular characteristics associated with social inequities and marginalisation (e.g., considering service users of migrant and ethnic minority backgrounds, and sexual and gender minority communities). Furthermore, there is a lack of data on the experiences of male survivors[20]; those with a disability even though they are at greater risk of sexual violence[21]; interactions between sexual violence exposure and chronic mental health problems, and co-morbidity of health outcomes; non-partner sexual violence[22] and sexual health outcomes other than the acquisition of sexually transmitted infections (STIs)[23].

This mixed-methods cohort study is the first to consider the health outcomes and cost trajectories of those who attend SARCs in England. It aims to evaluate the relationship between SARC factors, subsequent care (follow on specialist sexual assault services and

For peer review only - http://bmjopen.bmj.com/site/about/guidelines.xhtml

mental health care), and trajectories of PTS; the relationship between participant characteristics and trajectories of PTS; and finally, the role that participant characteristics may have in moderating the benefits of different SARC factors and subsequent care. It represents the first such study to consider the broader sexual health and wellbeing of survivors attending SARCs alongside other assessments of their health and mental wellbeing over time. We also report here how findings from our pilot study at one English SARC and our early data collection experiences have informed a revised research protocol.

METHODS AND ANALYSIS

Design and setting

This is a mixed-methods cohort study of mental, physical, sexual health and cost outcomes over one year in adult survivors of sexual assault and rape who have received care through SARCs in England. Quantitative measures are taken at baseline (no sooner than 4 weeks)) and at 6 and 12-months post-baseline. A nested qualitative study is included (see Qualitative study section below). The study is part of the MESARCH programme (September 1, 2018 to January 31, 2023) The wider programme includes two Cochrane Reviews[24 25], and 'mapping' English SARCs, their structure and services, in addition to the study described here.

Sampling, recruitment and procedures

We have recruited a stratified random sample of 15 of all the current practicing adult SARCs in England (47 identified at time of writing) with strata defined according to service delivery model, size and level of integration of services. To do this, we have drawn on national indicators of performance data collected by NHS England and NHS Improvement[14], the commissioning body for SARCs, and data collected from SARCs as part of the wider MESARCH study.

Recruitment is taking place in three phases. Firstly, service users aged 18 years and above presenting in person (or remotely since Covid-19) at any of the 15 participating SARCs are screened by SARC staff. People are excluded if in exercising judgement, the responsible member of SARC staff conducting screening anticipates they may encounter difficulties in providing informed consent due to severe mental health issues, cognitive impairment or intellectual disability. We also exclude those visiting from prison settings, as conducting follow-up assessments in prisons is outside the scope of the project. SARC staff complete a screen form for all those over 18 presenting to the service, recording basic demographic data, and offence and referral characteristics and indicating whether the person is eligible or not, with any reasons for exclusion noted. These screen forms are given an identification

For peer review only - http://bmjopen.bmj.com/site/about/guidelines.xhtml

number, contain no identifying information, and are passed to the project team to provide an indication of the characteristics of the pool of service users pre-enrolment.

Because many SARCs refer on quickly to other voluntary sector agencies (i.e., NGOs), for each participating SARC, we identified all linked support agencies and worked to engage them. The second phase of recruitment mainly takes place in that follow-on setting. When the person is referred to follow-on services, the identification number assigned at SARC travels with them. The study is introduced in that setting by trained staff from the agency, and if the person/service user expresses an interest in hearing more about the study, the staff member seeks consent to pass on contact details to the researchers. The identification number is also passed to the research team at this point, thus allowing the researchers to link the person/contact details with the SARC data obtained in the first phase.

In the final phase, the research team takes responsibility for contacting service users and inviting them to participate. Table 1 below provides further detail on the recruitment procedure. SARC service users may also self-refer into the study and be referred directly by SARC. Anyone aged 18 years or over who has visited a SARC in England during the recruitment phase (2 years) since 1st August 2019 was eligible for enrolment via direct contact with the research team. Please note a separate study focused on 13–17-year-olds is also being conducted as part of the wider MESARCH programme.

Number of participants required

At each of the 15 participating SARCs and via some self-referral, eligibility and basic sociodemographic data are collected on a total of 2500 individuals enabling us to compare our cohort sample with the wider population of service users at SARC. The primary outcome of interest for evaluating service user journeys after attending a SARC is post-traumatic stress (PTS), measured using the PCL-5[26]. Our interpretation of models based on the PCL-5 will focus on what constitutes a minimal clinically important difference (MCID) for PTS. Stefanovics and colleagues[27] concluded that differences of approximately 0.5 of a standard deviation (SD) could be deemed clinically important both cross-sectionally and for within-person change. Since our study involves neither randomisation nor placebo-control, our analyses will focus on change in PTS symptoms between baseline and follow-up. We will take recommendations of Stefanovics et al.[27] as quantifying the change-score group-difference we would expect which separates a treatment that is effective from one that is ineffective. Based on guidelines[26], we anticipate this MCID of 0.5 SD to approximate to a 10-point difference on the PCL-5 change-score; however, Stefanovics et al.[27] propose that an SD-based approach would be more robust and we also recognise that the magnitude of

For peer review only - http://bmjopen.bmj.com/site/about/guidelines.xhtml

change in on the raw-scale metric will be influenced by the length of time between baseline and follow-up.

Table 1. Procedure for involving adult service users in the MESARCH cohort study

1. *Service user receives care from SARC.*
2. *SARC staff member screens service user for eligibility. All eligibility information is de-identified (assigned an identification number only) and conveyed to the project team using secure sharing platform.*
3. *A referral is made by SARC to an Independent Sexual Violence Advisor (ISVA; usually based within a voluntary sector agency) for follow-on care, in line with usual practice, and the research identification number is placed on the referral form. If the service user is not willing to be referred to other agencies, the SARC could provide more information about the study at this point.*
4. *Within the voluntary sector agency, eligible service users are approached mainly by ISVAs, who provide brief explanation about the project and invite service users to consider being involved in the study. For those who express an interest in hearing more, consent is requested to pass contact details to the project team and the person's research identification number is also included ('Level 1' consent completed).*
5. *Additional approaches for enabling recruitment include (i) having members of the project team ready to speak by phone or video link to a service user if the SARC worker/ISVA believes this is an appropriate approach for a particular service user; (ii) showing a short video message of invitation co-produced with our Lived Experiences Group which could be used by SARC staff/ISVA to explain about the project (available on our project website and accessible using a QR code on study brochure); (iii) opening study up to self-referrals by survivors who have used SARCs in England during the study recruitment phase.*
6. *Once the contact information has been passed on to the researchers, there is no more involvement of the SARC/ISVA except in circumstances such as the participant requires support and has agreed for us to notify the SARC/ISVA.*
7. *A trained project team member makes contact within 1 week of receiving the research referral consent form from the SARC or ISVA, and contact is made in line with service user preferences for example by email, text or phone call.*
8. *Once contact is made by the project team at baseline, the project team will follow recruitment and safety protocols, explain study purpose and gain full consent ('Level 2' consent).*
9. *When consent to take part in the study is established (using email or text or signed consent form), baseline data are collected using a range of options. These include offering a structured telephone interview, a weblink to complete the data collection online, an interview in person or via video link. Those submitting baseline data will be considered 'enrolled' in MESARCH.*
10. *Follow-up is undertaken according to the participants' preferences at 6 and 12 months consistent with our previous work*[28]

For peer review only - http://bmjopen.bmj.com/site/about/guidelines.xhtml

To allow for 4 levels in a predictor variable (e.g. 4 types of SARC service provider); up to 10 covariates; two time points; an expected small-to-moderate Cohen's f, which corresponds to the minimum clinically important difference (MCID) for our primary outcome variable[27]; and an α error probability of 0.05, 270 participants would be required at study end to achieve a power (1-β error probability) of 0.90. Based on previous studies,[18 19] our pilot work, and recruitment and retention to date, we estimate attrition at 20% by 12-month follow-up, requiring a baseline target of 338 individuals (see estimated flow of participants in Figure 1). Participants who do not complete a 6-month follow-up will still be eligible to complete 12-month follow-up measures.

Figure 1 about here

Data collection

The form used by SARC staff to screen service users for the research generates data on gender; age; ethnicity; offence characteristics; referral into SARC; and indicators of vulnerability at point of service access (e.g. history of mental health problems, disability, substance use). At baseline, we collect additional detailed socio-demographic information on marital/relationship status; nationality; immigration status; number of children; sexual identity; disability; religion; education; employment status and income; and accommodation. Baseline data is gathered by structured interviews, by telephone, video link or in-person by trained research staff who enter responses directly into secure bespoke study software linked to Qualtrics survey software. We enquire about adverse childhood experiences using the WHO ACE International Questionnaire and screen for lifetime and past year domestic abuse using the ACTS[29] to enable us to describe the sample. All other baseline measures focus on standardised timeframes according to the measure, or the period since participants attended the SARC. Follow-up is taking place on two occasions over the course of the project, at 6 months and 12 months. Our proposed methods for retaining participants are informed by a number of large studies of violence and health[30] including our own previous work in the health field[31]. These include gathering a range of contact details from participants at baseline - safe telephone numbers; postal/email addresses; use of reminders; and providing a small incentive (i.e., £10 following baseline completion, £10 at 6 months and £15 at 12 months).

For peer review only - http://bmjopen.bmj.com/site/about/guidelines.xhtml

For peer review only

Outcome	Measure*	Timepoint
PTS	PCL-5	Baseline, 6 months, 12 months
Depression	CESD-R-10	Baseline, 6 months, 12 months
Quality of life	WHOQoL-Bref	Baseline, 6 months, 12 months
Eating issues	SCOFF<br>BEDS-7	Baseline, 6 months, 12 months
Health related quality of life	EQ5D-5L	Baseline, 6 months, 12 months
Capability	ICEpop CAPability measure for Adults (ICECAP-A)	Baseline, 6 months, 12 months
Sexual health	Bespoke measure	Baseline, 6 months, 12 months
Alcohol use	AUDIT-C	Baseline, 6 months, 12 months
Drug use	DUDIT	Baseline, 6 months, 12 months
Resource use	Bespoke measure	Baseline, 6 months, 12 months
Adverse Childhood Experience	WHO ACE International Questionnaire	Baseline
Intimate Partner Violence	ACTS 4 item screen<br>CAS	Baseline<br>6 months, 12 months

Outcomes

Table 2 shows the measures at each time point. The primary outcome is post-traumatic stress (PTS), widely endorsed in the literature [4 9] and through our scoping work with individuals with lived experience and service providers, as a primary health issue for survivors and an absence of diagnosis, or reduction in symptoms may mark improvement or recovery in a person who has experienced sexual violence. Our analyses will focus on change or improvement in PTS symptoms between baseline and follow-up. We have developed a set of items to assess sexual health, including STI diagnosis; pregnancy and pregnancy outcomes, and reproductive coercion[32 33]. Though we screen for sexual violence re-exposure at follow-up, discussion of the experience of sexual violence that led the person to seek help at the SARC is not encouraged in order to protect victims' testimonies and to create a safe space for the person to participate in the research. Our approach to measuring health and other costs using the ICECAP-A[34] and EQ5D-5L[35] is outlined in *Economic Evaluation* below. We have also developed a comprehensive evaluation of SARC service users' onwards service utilisation and satisfaction with care that considers the many sectors that are implicated in meeting the needs of survivors in the aftermath of an assault or abuse.

For peer review only - http://bmjopen.bmj.com/site/about/guidelines.xhtml

Sexual violence	Bespoke measure	6 months, 12 months
Suicidality and self-harm	Items from the Adult Psychiatric Morbidity Survey	Baseline, 6 months, 12 months

Table 2. Outcome measures and the timepoint at which they are collected.

Patient and public involvement (PPI)

The funding application for this research was made in response to a commissioned call from the National Institute for Health Research (NIHR) which set out a series of research questions concerning the evaluation of SARCs in England. We met with survivors of sexual violence as well as other stakeholders (e.g., rape crisis services, SARC staff) and consulted them throughout the funding application process to ensure that the proposed research met the diverse needs of survivors and providers, and was acceptable and feasible from their perspectives. When funding was awarded, we formalised PPI by recruiting a group of survivors to support the study on an ongoing basis. Members named the PPI group as the Lived Experiences Group (LEG). and it is made up of ten survivors from different backgrounds and experiences. Two members of the LEG sit on our study steering committee (SSC) and the wider group has been involved in every aspect of our study decision-making, the design of our materials, our communications about the study and production of outputs, development of follow-on projects. The LEG will continue to support the process of understanding and interpreting our findings, shaping the recommendations we make, and disseminating effectively with all stakeholders, including our participants, about what we find.

Data analysis

Our analysis will determine if variation in SARC service delivery and other healthcare specific to sexual violence is associated with different levels of improvement in trauma symptoms between baseline and the 12-month follow-up. Our favoured statistical approach will be to model "trajectories" of PTS using mixed-effects regression (i.e., Multilevel Modelling, using Stata's suite of mixed-effects tools[36]). This framework will enable us to model variation in the improvement in PTS and make best use of the three waves of data available. In the eventuality that change appears non-linear, we will firstly explore the utility of incorporating varying times of follow-up and modelling time-since-baseline rather than data-collection-wave as the time axis, although we will be mindful of the potential for introducing bias by incorporating such a source of variation. We will also consider standard single-level regression models (e.g., OLS regression, ANCOVA) and model observed

For peer review only - http://bmjopen.bmj.com/site/about/guidelines.xhtml

change in PTS between baseline and 12-months, instead using the 6-month wave to assist with the treatment of any missing data using multiple imputation. In addition, and where appropriate, we will accommodate the nested data-structure of participants within SARC-centres using cluster-robust standard errors. Similar analyses will be carried out for the secondary outcomes.

(1) Following the investigation of the optimal “unconditional” model to describe changes in PTS and the other outcomes of interest, we will introduce SARC-level characteristics (whether ISVAs are located at SARC or externally-provided; whether the SARC is run by police, NHS, voluntary agency or private company; regional capacity for meeting survivors' needs; ease of access to SARC) and characteristics related to follow-on care for sexual violence and mental health (uptake of ISVA care, access to therapy in the NHS or elsewhere), as a series of fixed effects. (2) The next stage of analysis will seek to address the question ‘How do trajectories compare for different subgroups of survivors attending SARCs?’ In the event that the mixed models described above can be empirically justified, we will estimate outcome trajectories for different groups incorporating recent v non-recent victimisation including time lag between trauma and SARC visit, ACEs, gender, sexuality, culture, ethnicity, disability and chronic mental health problems and perceived benefit/harm from services. Where necessary, the single-level equivalent for this analysis stage will examine whether mean differences in PTS improvement vary across the same participant characteristics. Whilst this stage mentions “subgroups”, and technically involves an interaction (with time since baseline), this can be considered both conceptually, and in terms of statistical power, to be a series of main-effects analyses. (3) Finally, we will investigate the role that participant characteristics may have in moderating the benefits of different SARC factors and follow-on care for sexual violence and mental health. This analysis will be both exploratory and pragmatic given the likely low power for this step and will be driven by the findings from stages (1) and (2) above. We will focus on those minority groups large enough to enable robust statistical comparison and formal interactions tests to be performed. In instances where some subgroups of interest are small (e.g. male survivors), hindering formal statistical tests, we will take a more descriptive approach and be cautious about our conclusions.

Economic evaluation

Design

For peer review only - http://bmjopen.bmj.com/site/about/guidelines.xhtml

The economic analysis will compare the costs and outcomes associated with different organisational aspects of SARC service delivery. If some SARC models are more strongly associated with reducing PTS and improving quality of life and other mental, physical and sexual health outcomes than others, there are likely to be important cost implications for the healthcare sector, for the wider public sector, and for society as a whole.

Data collection

Resource use data will be collected prospectively to estimate the costs associated with different models of SARC service delivery. The resource use to be monitored will include: 1) the cost of service use within SARCs (e.g. consultations, treatment etc.); 2) NHS and other public sector resource use after initial attendance at SARCs (e.g. GP visits, sexual health visits); 3) costs associated with the treatment of PTS and other relevant conditions; 4) wider public sector resource use, for example in relation to social care, housing, and other social welfare systems; 5) costs experienced by service users and family members. Information on unit costs or prices will be sourced to attach to each resource use item (e.g. [37]). Health-related quality of life data will be collected using the EQ5D-5L instrument which is widely used for those with PTSD and related conditions [38].

Economic analysis

In order to compare the costs and benefits of different SARC service delivery models, both a within study analysis and a model based economic analysis will be undertaken. The main economic analysis will assess cost-effectiveness based on incremental cost per quality-adjusted life year (QALY) gained at 6 and 12 months, with a secondary analysis of cost per case of PTS avoided at 12 months. If the results of the cohort study show that there are significant differences in the effectiveness of SARC delivery models, in terms of reducing PTS and improving other health outcomes, it will be necessary to assess the cost-effectiveness of the SARC delivery models in the longer term, to take into account the impact on an individual's quality of life and productivity. Therefore, if deemed necessary, based on the results of the cohort study, we will use a decision-analytic model to evaluate the longer-term impacts of the different types of service delivery (for up to five years, if data allow). The model development process will use, as a starting point, other models developed for PTS and related conditions (e.g. [39]). We will use both deterministic and probabilistic sensitivity analyses to explore the effects of inherent uncertainty in the estimates on the results [40]. For the longer-term analyses, discounting will be undertaken to reflect recommendations by NICE [41].

Pilot study

For peer review only - http://bmjopen.bmj.com/site/about/guidelines.xhtml

A pilot study was conducted with baseline recruitment over three months between April and June 2019 at one SARC site. The purpose was to examine the feasibility of the methods for the main cohort study and identify required design modifications. We assessed the feasibility of our approach to inviting participants into the study, data gathering procedures, and retention (e.g., rates and use of incentives). At the SARC, we discussed the project with staff members, built awareness about the rationale, explained inclusion/exclusion criteria and use of the screening form to record eligibility, and agreed on the most appropriate staff members to approach potential participants at different stages. Once set-up was complete, all eligible service users over a 3-month period were invited into the pilot project by SARC workers. The pilot included telephone interviews at baseline and one follow-up only, at 3 months. Data collection included a set of questions about the experience of being invited into and participating in the study.

Over three months, 43 service users attended the pilot SARC (classed as small relative to other English SARCs). Staff tended to be highly conservative in applying the eligibility criteria, voicing concerns about mental ill-health and overburdening the service users (n=15). Of the 28 service users identified as eligible, 11 lost contact with the SARC prior to being invited into the study and three declined to have their contact details passed on. Of the 14 people who agreed to be contacted by the research team, four could not be contacted and ten were successfully enrolled. From those ten participants, there were no missing data and retention at 3 months was 100%. We encouraged think aloud responses to items and requested feedback about people's experiences of the pilot. These were all positive comments. For example, *"I really enjoyed being a part of the study. It felt great to be able to take an unfortunate event and turn it into something positive and it's nice knowing that what I contributed will go on to help others" (23yr old female participant in pilot study*).

We are aware from another similar study,[18 19] our own pilot study and our early recruitment experiences of the challenges in recruitment. For the main study, we are providing clearer guidance to staff about the exceptional circumstances under which a person should be identified as ineligible. We are aware of the circumstances of people's lives that make research participation difficult, but we believe they should, wherever possible, be given full choice around being involved or not. Rates of exclusion at the SARC level are continuously monitored during recruitment and further training and communication interventions are implemented where deemed necessary. We have continued to focus our efforts on minimising the numbers of individuals who are potentially 'lost' between SARC and the follow up care in NGOs and other settings, prior remains our primary and safest avenue for reaching potential participants. In addition to working with ISVAs, we have cascaded information across other practitioners in the voluntary sector (e.g. counsellors) and sexual

For peer review only - http://bmjopen.bmj.com/site/about/guidelines.xhtml

health services to promote self-referral. We regularly refer participants and potential participants to our up to date website where people can read about the origins and aims of the work; understand about the people carrying out the work, including the role of our lived experiences group members; and access testimonials from other survivor participants. We offer options to participants wherever possible (e.g. choice of gender of interviewer and speaking with a peer researcher; offering interview times out of hours and doing interview in parts if desired; providing overview of each section before any questions are asked enabling people to skip sections/questions; providing resources and follow-up where concerns arise). We expect such measures will enhance our retention and minimise missing data.

Qualitative study

Design

There will be a nested qualitative, interview-based study to enable a greater depth of understanding of the factors associated with better outcomes for survivors of sexual violence participating in the adult cohort study. It will include a community-based comparison sample. We will include around 50 individuals aged 18 years and above.

The study will employ narrative methods [42] as these provide participants with the opportunity to give their accounts of a particular experience, free of the assumptions of the interviewer or research team and empowering them to structure the stories of what happened, and their meanings as understood by them. Narrative methods have been applied extensively in explorations of experiences relevant to SV and rape (e.g., [43-45]) and lend themselves well to gaining insight into the ways in which people get to grips with potentially devastating disruptions to their everyday lives. For example, Becker's[46] work takes an in-depth narrative approach to considering experiences of five people who have had different major disruptive life events including illness and infertility[46].

Recruitment of participants

Participants who have participated in the adult cohort study will be recruited in order to gain insight into factors that were experienced positively and negatively from a range of narratives. This will provide the project team with rich data about experiences outside of SARC models of care that were valued, as well as those within SARC models. In collaboration with our Lived Experiences Group and professionals who support survivors, we will devise a sampling framework and detailed recruitment strategy that is sensitive to the needs of, and acceptable to, the target population. We propose that this will involve recruiting adults from the SARC cohort study after their 12-month measures are collected.

For peer review only - http://bmjopen.bmj.com/site/about/guidelines.xhtml

For peer review only

We will aim to recruit around 30 cohort members in total and apply maximum variation sampling in order to over-represent service users whose voices are not typically included in this type of research (e.g. in this context, gender, sexual, age and ethnic minorities; participants affected by issues such as homelessness, sex work, disability and long-term mental health difficulties).

Recruitment of non-SARC service users

We will also seek to recruit people via voluntary sector partners and stakeholders who support survivors of sexual violence. This will ensure wider experiences are included in the data (approximately 20 individuals) and will help the research team to better understand barriers to SARC access among those offered a referral by other services but who have not used SARCs.

Procedure

Participants who express an interest in being interviewed will be given time and support to prepare for the narrative interview having had a full explanation of what this involves and ensuring they are comfortable to provide their story about experience of services following SV. Participants will be offered the choice of a face-to-face (where Covid-19 restrictions allow), online video conference, or telephone interview at a time and location convenient to them and either with or without the support of a person of their choosing. The interview guide will be developed in partnership with survivors of sexual violence and those who support them within our lived experience group. It is likely to include an opportunity for the participants to talk generally about themselves and how they are, and to discuss any concerns they may have about the narrative interviews they are engaging with. The interviewer will discuss any concerns and ensure that the participant is happy to talk about their recovery journey. The interviewer will likely suggest the participant starts at an appropriate moment in discussions by saying that he/she/they are, 'interested in finding out about your experiences of accessing help or support and the impact on your health and wellbeing, after experiencing sexual violence' and, 'you can begin to tell us your experiences of receiving support and your recovery journey so far, at the point where you first sought help in relation to your experience of sexual assault, or wherever it feels right to start'. We will be clear through our participant information sheet and consent procedures that we are not asking participants to talk about the incident(s) that led them to seek help and support. Interviews will be audio-recorded with participants' permission and transcribed verbatim.

Analysis of qualitative data

For peer review only - http://bmjopen.bmj.com/site/about/guidelines.xhtml

For peer review only

Data will then be subject to narrative analysis [42] to draw out the meanings ascribed to participants' experiences and to identify both unique elements and commonality or themes across experiences. This process will be led by KB, with support from GC and another research assistant. Narrative analysis is divided into two distinct phases. The first is a descriptive phase which, following thorough reading and familiarisation with the transcripts, involves devising summaries of each narrative to pull out the key features and identify sub-plots as well as overarching story arcs. Similarity between different narrative summaries, as well as key differences, will also be identified at this stage to form the basis of a coding frame. In the second stage, a range of theoretical perspectives will be considered in order to interpret and make sense of the narratives and the coding frame. To achieve this we will work collaboratively with co-investigators and Lived Experience Group members, considering the range of options and the ways in which they may or may not aid interpretation of the data. This process will be used to support interpretation of quantitative findings from the cohort data.

ETHICS AND DISSEMINATION

The pilot study and main cohort study have received ethical approval from Coventry University (pilot: P76598; main study: P86669) and NHS Research Ethics (pilot: 18/WM/0376; main study: 19/EM/0198). NHS Health Research Authority approval was also provided before data collection began.

The research is supported by an independent study steering committee (SSC), a data monitoring and ethics committee (DMEC) and a Patient and Public Involvement (PPI) group, known as the Lived Experiences Group (LEG – see Patient and Public Involvement section above The SSC has been in place since study set up and is made up of a range of relevant academic, government, non-government and independent agencies and experts who can offer guidance and support on the challenges of conducting the research throughout. One example of SSC and LEG input includes working with them to refine our choice of measures, in particular, appropriate ways to assess sexual violence exposure in this high-risk population [47]. A further example is having worked with some of our LEG members on the development of short videos to introduce the study to professionals and survivors to support recruitment. Both of these films can be accessed on the study website at http://mesarch.coventry.ac.uk/getting-involved/. The DMEC is meeting three times throughout the cohort study (baseline, post 6 months FU and post 12 months FU) to assess data, analyses, and whether any evidence of harm should lead to a recommendation that the study ceases.

For peer review only - http://bmjopen.bmj.com/site/about/guidelines.xhtml

The experience, safety and wellbeing of survivors, participants and staff is of paramount importance in this research. All staff involved in delivering this cohort study and the wider research activities in the funded project have undergone specialist training from two voluntary sector organisations who work to support survivors of sexual violence, participate in a weekly team meeting and receive regular training and external clinical supervision. We have developed a detailed safety protocol to define clearly what the actions of staff should be if there are any safety, safeguarding or wellbeing concerns raised during research procedures. These are with regards to potential participants, participants and any children or other dependents, and staff in line with NIHR Policy on Preventing Harm in Research. A central guiding principle of the research is that being involved as a participant should feel like the opposite of being a victim of sexual violence, derived from by The Survivors' Charter developed by Survivors' Voices[48]. In other words, it should involve feeling empowered, respected, and in control at all times. In fact, in our experience of data collection to date, during the pilot and the first participants at baseline in the main cohort study, people report exactly that. They are happy to and want to participate in the research because it is an opportunity for them to pro-actively contribute something positive to improve services in the future. It is their decision to do so and they are in control of the answers and information they provide.

One issue that it has been particularly pertinent to consider is the possibility that data provided by participants involved in an investigation or pursuing a prosecution may be subject at any time to subpoena by the courts or requested by law enforcement. Participants are made aware of this before providing consent, and the focus of the research is not what happened during any alleged incident of sexual violence. Our safety protocol involves a requirement that staff remind participants about the limits of confidentiality at each time point, including subpoena of interview notes. The team is regularly trained in how to manage disclosure of new information pertaining to a case or which signals a risk to the welfare of a child or vulnerable adult.

Dissemination of the findings will be supported by our SSC and the LEG through a range of channels in the future. In addition to standard publication in peer reviewed academic journals and at academic conferences, we will present findings at policy and practice-based conferences nationally and internationally. We will publish findings in lay language on our study website and produce other forms of lay publications targeted at all agencies and organisations with a remit for addressing sexual violence and its consequences in society. We will work with NHS England who commissioned the research to ensure the findings are translated appropriately into policy and practice.

For peer review only - http://bmjopen.bmj.com/site/about/guidelines.xhtml

Author contributions: LOD conceived the study design with support and input from GF, RM, RC, LJ and KB. RM paid particular attention to designing the analysis plan and LJ to designing the health economics plan. LOD led the successful application for funding with contributions from GF, RM, RC, LJ and KB. As study coordinator, GC supported by E L-W are overseeing recruitment and data collection and are managing the preparation of data in readiness for analysis. RM drafted the original cohort study analysis plan which was updated by JH. GC leads the children and young people's study. KB leads the nested qualitative study. KB drafted the manuscript based on the approved study protocol V3.3 and all co-authors read, contributed to re-drafting and approved the submitted manuscript. All authors have agreed to be accountable for all aspects of the work.

FUNDING STATEMENT

This work is supported by the National Institute for Health Research (Health Services & Delivery Research programme) grant number 16/117/04. The views expressed are those of the authors and not necessarily those of the NIHR or the Department of Health and Social Care.

COMPETING INTERESTS STATEMENT

The authors have no competing interests to declare

ACKNOWLEDGEMENTS

We acknowledge the contributions of Millie Gant, Juniper Lodge SARC; Dianne Whitfield, Rape Crisis England, Priya Tek-Kelsi, the project PPI co-ordinator for her significant contribution to getting this work off the ground; and all the members of our lived experience group, the study steering committee and the data monitoring and ethics committee for all their contributions and support.

Word count: 5747 words

For peer review only - http://bmjopen.bmj.com/site/about/guidelines.xhtml

References

1. ONS. Violent crime and sexual offences-Intimate personal violence and serious sexual assault., 2015.
2. Krug EG, Mercy JA, Dahlberg LL, et al. The world report on violence and health. *The lancet* 2002;360(9339):1083-88.
3. ONS. Sexual offending: victimisation and the path through the criminal justice system, 2018.
4. World Health Organization. Global and Regional Estimates of Violence Against Women: Prevalence and Health Effects of Intimate Partner Violence and Non-Partner Sexual Violence. Geneva (CH): World Health Organization 2013.
5. Campbell JC. Health consequences of intimate partner violence. *The lancet* 2002;359(9314):1331-36.
6. Tewksbury R. Physical, Mental and Sexual Consequences. *International Journal of Men's Health* 2007;6(1):22-35.
7. Coxell AW, King MB. Male victims of rape and sexual abuse. *Sexual and Marital Therapy* 1996;11(3):297-308.
8. Walker J, Archer J, Davies M. Effects of rape on men: A descriptive analysis. *Archives of sexual behavior* 2005;34(1):69-80.
9. Pietrzak RH, Goldstein RB, Southwick SM, et al. Prevalence and Axis I comorbidity of full and partial posttraumatic stress disorder in the United States: results from Wave 2 of the National Epidemiologic Survey on Alcohol and Related Conditions. *Journal of Anxiety Disorders* 2011;25(3):456-65.
10. Liu H, Petukhova MV, Sampson NA, et al. Association of DSM-IV posttraumatic stress disorder with traumatic experience type and history in the World Health Organization World Mental Health Surveys. *JAMA psychiatry* 2017;74(3):270-81.
11. Guina J, Nahhas RW, Kawalec K, et al. Are gender differences in DSM-5 PTSD symptomatology explained by sexual trauma? *Journal of interpersonal violence* 2019;34(21-22):4713-40.
12. Dubourg R, Hamed J, Thorns J. The economic and social costs of crime against individuals and households 2003/04. *Home Office online report* 2005;30(05)
13. Organization WH. Guidelines for medico-legal care of victims of sexual violence. 2003
14. England N. Public Health Functions to be Exercised by NHS England–Service Specification: Sexual Assault Referral Centres. *NHS England* 2018
15. England N. Sexual Assault and Abuse no date [Available from: https://www.england.nhs.uk/commissioning/health-just/sexual-assault-and-abuse/#sarcs accessed 09/03/21 2021.
16. Lovett J, Regan L, Kelly L. Sexual Assault Referral Centre s : developing good practice and maximising potentials2004.
17. Abrahams N, Mhlongo S, Dunkle K, et al. Increase in HIV incidence in women exposed to rape. *AIDS (London, England)* 2021;35(4):633.
18. Abrahams N, Seedat S, Lombard C, et al. Study protocol for a longitudinal study evaluating the impact of rape on women’s health and their use of health services in South Africa. *BMJ open* 2017;7(9)
19. Abrahams N, Mhlongo S, Chirwa E, et al. Rape survivors in South Africa: analysis of the baseline socio-demographic and health characteristics of a rape cohort. *Global Health Action* 2020;13(1):1834769. doi: 10.1080/16549716.2020.1834769
20. Weare S, Hulley J, Pattinson B. A Review of the National Male Survivors Helpline and Online Service-Project Report: Lancaster University, 2019.
21. Basile KC, Breiding MJ, Smith SG. Disability and risk of recent sexual violence in the United States. *American journal of public health* 2016;106(5):928-33.
22. Ellsberg M, Arango DJ, Morton M, et al. Prevention of violence against women and girls: what does the evidence say? *The Lancet* 2015;385(9977):1555-66.

For peer review only - http://bmjopen.bmj.com/site/about/guidelines.xhtml

23. O'Driscoll C, Flanagan E. Sexual problems and post-traumatic stress disorder following sexual trauma: A meta-analytic review. *Psychology and Psychotherapy: Theory, Research and Practice* 2016;89(3):351-67.
24. Brown SJ, Khasteganan N, Brown K, et al. Psychosocial interventions for survivors of rape and sexual assault experienced during adulthood. *Cochrane Database of Systematic Reviews* 2019(11) doi: 10.1002/14651858.CD013456
25. Brown SJ, Khasteganan N, Carter GJ, et al. Survivor, family and professional experiences of psychosocial interventions for sexual abuse and violence: a qualitative evidence synthesis. *Cochrane Database of Systematic Reviews* 2020
26. Checklist P-P. [Available from: https://www.ptsd.va.gov/professional/assessment/adult-sr/ptsd-checklist.asp accessed 23rd September 2018.
27. Stefanovics EA, Rosenheck RA, Jones KM, et al. Minimal clinically important differences (MCID) in assessing outcomes of post-traumatic stress disorder. *Psychiatric Quarterly* 2018;89(1):141-55.
28. Hegarty KL, Gunn JM, O'Doherty LJ, et al. Women's evaluation of abuse and violence care in general practice: a cluster randomised controlled trial (weave). *BMC Public Health* 2010;10(1):1-11.
29. Hegarty K, Spangaro J, Kyei-Onanjiri M, et al. Validity of the ACTS intimate partner violence screen in antenatal care: a cross sectional study. *BMC Public Health* 2021;21(1):1733. doi: 10.1186/s12889-021-11781-x
30. Ford-Gilboe M, Varcoe C, Scott-Storey K, et al. A tailored online safety and health intervention for women experiencing intimate partner violence: the iCAN Plan 4 Safety randomized controlled trial protocol. *BMC Public Health* 2017;17(1):1-12.
31. Hegarty K, O'Doherty L, Taft A, et al. Screening and counselling in the primary care setting for women who have experienced intimate partner violence (WEAVE): a cluster randomised controlled trial. *The Lancet* 2013;382(9888):249-58. doi: https://doi.org/10.1016/S0140-6736(13)60052-5
32. Grace KT, Anderson JC. Reproductive Coercion: A Systematic Review. 2018;19(4):371-90. doi: 10.1177/1524838016663935
33. Maxwell L, Devries K, Zionts D, et al. Estimating the effect of intimate partner violence on women's use of contraception: a systematic review and meta-analysis. *PloS one* 2015;10(2):e0118234.
34. Al-Janabi H, Flynn TN, Coast J. Development of a self-report measure of capability wellbeing for adults: the ICECAP-A. *Quality of life research* 2012;21(1):167-76.
35. EQ5D ERF. EQ-5D-5L [Available from: https://euroqol.org/eq-5d-instruments/eq-5d-5l-about/ accessed 1st September 2019.
36. Stata. Multilevel Mixed-Effects Reference Manual: Stata Press 2021.
37. Curtis L, Burns A. Unit Costs of Health and Social Care. Canterbury: Personal Social Services Research Unit, University of Kent 2015.
38. Ophuis RH, Lokkerbol J, Heemskerk SC, et al. Cost-effectiveness of interventions for treating anxiety disorders: A systematic review. *Journal of affective disorders* 2017;210:1-13.
39. Mihalopoulos C, Magnus A, Lal A, et al. Is implementation of the 2013 Australian treatment guidelines for posttraumatic stress disorder cost-effective compared to current practice? A cost-utility analysis using QALYs and DALYs. *Australian & New Zealand Journal of Psychiatry* 2015;49(4):360-76.
40. Briggs AH, Weinstein MC, Fenwick EA, et al. Model parameter estimation and uncertainty: a report of the ISPOR-SMDM Modeling Good Research Practices Task Force-6. *Value in Health* 2012;15(6):835-42.
41. Woolf SH, Purnell JQ, Simon SM, et al. Translating Evidence into Population Health Improvement: Strategies and Barriers. *Annual Review of Public Health* 2015;36(1):463-82. doi: 10.1146/annurev-publhealth-082214-110901
42. Murray M. Narrative Psychology in Qualitative Psychology In: Smith JA, ed. Qualitative psychology: A practical guide to research methods: Sage Publications, Inc 2003.

For peer review only - http://bmjopen.bmj.com/site/about/guidelines.xhtml

43. Anderson S, Jia JX, Liu V, et al. Violence prevention and municipal licensing of indoor sex work venues in the Greater Vancouver Area: narratives of migrant sex workers, managers and business owners. *Culture, health & sexuality* 2015;17(7):825-41.
44. Kim T, Draucker CB, Bradway C, et al. Somos Hermanas Del Mismo Dolor (We are sisters of the same pain) Intimate partner sexual violence narratives among Mexican immigrant women in the United States. *Violence against women* 2017;23(5):623-42.
45. Foster JM, Hagedorn WB. Through the eyes of the wounded: A narrative analysis of children's sexual abuse experiences and recovery process. *Journal of child sexual abuse* 2014;23(5):538-57.
46. Becker G. Disrupted lives: How people create meaning in a chaotic world: Univ of California Press 1997.
47. Probst DR, Turchik JA, Zimak EH, et al. Assessment of sexual assault in clinical practice: Available screening tools for use with different adult populations. *Journal of Aggression, Maltreatment & Trauma* 2011;20(2):199-226.
48. Voices S. The Survivors' Charter [Available from: https://survivorsvoices.org/charter/#:~:text=Turning%20Pain%20into%20Power%3A%20A,London%20and%20the%20Wellcome%20Foundation.

For peer review only

Figure caption for figure 1.

Figure 1. Flowchart of service users aged 18 years and above in the MESARCH cohort study

For peer review only - http://bmjopen.bmj.com/site/about/guidelines.xhtml

Figure 1. Flowchart of service users aged 18 years and above in the MESARCH cohort study

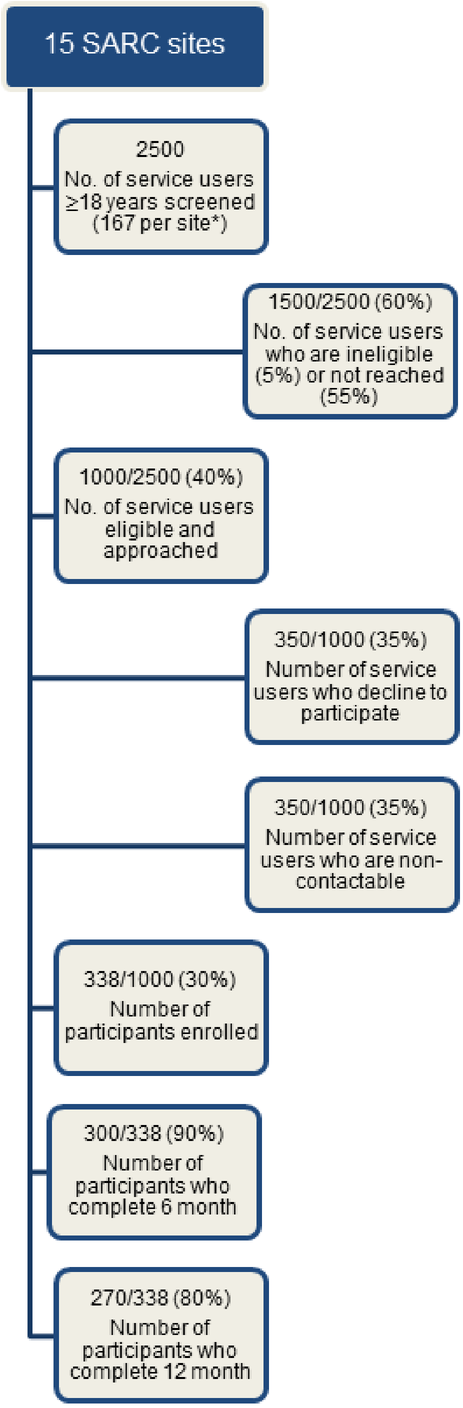

Supplement: Author's manuscript [file bmjopen-2021-057449.draft_revisions.pdf]
